# Epigenetic targets to enhance antitumor immune response through the induction of tertiary lymphoid structures

**DOI:** 10.3389/fimmu.2024.1348156

**Published:** 2024-01-25

**Authors:** Quadri Ajibola Omotesho, Alejandro Escamilla, Elisabeth Pérez-Ruiz, Cecilia A. Frecha, Antonio Rueda-Domínguez, Isabel Barragán

**Affiliations:** ^1^ Medical Oncology Service (Group of Translational Research in Cancer Immunotherapy and Epigenetics), Regional and Clinical University Hospitals, Instituto de Investigación Biomédica de Málaga y Plataforma en Nanomedicina-IBIMA Plataforma BIONAND, Malaga, Spain; ^2^ Department of Medical Biochemistry and Microbiology, Uppsala University, Uppsala, Sweden; ^3^ Department of Human Physiology, Human Histology, Pathological Anatomy and Physical Sport Education, University of Malaga, Malaga, Spain; ^4^ Allergy Research Group, Instituto de Investigación Biomédica de Málaga y Plataforma en Nanomedicina-IBIMA Plataforma BIONAND, Civil Hospital, Malaga, Spain; ^5^ Group of Pharmacoepigenetics, Department of Physiology and Pharmacology, Karolinska Institutet, Stockholm, Sweden

**Keywords:** epigenetics, viral vectors, B cells, CXCL13 chemokine, CXCR5, immunotherapy, biomarker, tertiary lymphoid structures

## Abstract

Tertiary lymphoid structures (TLS) are ectopic lymphoid aggregates found in sites of chronic inflammation such as tumors and autoimmune diseases. The discovery that TLS formation at tumor sites correlated with good patient prognosis has triggered extensive research into various techniques to induce their formation at the tumor microenvironment (TME). One strategy is the exogenous induction of specific cytokines and chemokine expression in murine models. However, applying such systemic chemokine expression can result in significant toxicity and damage to healthy tissues. Also, the TLS formed from exogenous chemokine induction is heterogeneous and different from the ones associated with favorable prognosis. Therefore, there is a need to optimize additional approaches like immune cell engineering with lentiviral transduction to improve the TLS formation *in vivo*. Similarly, the genetic and epigenetic regulation of the different phases of TLS neogenesis are still unknown. Understanding these molecular regulations could help identify novel targets to induce tissue-specific TLS in the TME. This review offers a unique insight into the molecular checkpoints of the different stages and mechanisms involved in TLS formation. This review also highlights potential epigenetic targets to induce TLS neogenesis. The review further explores epigenetic therapies (epi-therapy) and ongoing clinical trials using epi-therapy in cancers. In addition, it builds upon the current knowledge of tools to generate TLS and TLS phenotyping biomarkers with predictive and prognostic clinical potential.

## 1 Introduction

The classification of solid tumors into immunogenic “hot” and non-immunogenic “cold” types has revolutionized translational cancer research (1). Hot tumors have a characteristic tumor microenvironment (TME) enriched with tertiary lymphoid structures (TLS) and tumor-infiltrating lymphocytes (TILs). They typically present genomic instability and active immune interactions (2). On the other hand, the TME of cold tumors lacks these features. Hot tumors have been associated with significant treatment response and favorable prognosis in cancer therapy, while resistance and disease progression are related to a non-inflamed, cold tumor phenotype (3). Since the immunogenicity of solid tumors strongly determines therapeutic success, different methods have been employed to enhance the immune infiltration of the TME (4). For example, direct infusions of engineered chimeric antigen receptors (CAR) T-cells or TILs stimulated T-cell anti-tumor response within the TME (5). However, the application of CAR T-cells and TILs is limited by the absence of specific antigen targets and induced exhaustion of T-cells (6). Immune checkpoint inhibitors (ICI) blocking T-cells’ “off” switch have also been developed to enhance tumor infiltration and boost the immune response against cancer cells (7, 8). However, immunosuppressive interactions within the TME limit ICI function, resulting in resistance to immunotherapy and patient relapse (1, 9). Due to these limitations, newer approaches to modulate the TME are being considered. Recent studies have identified that the presence of tertiary lymphoid structures (TLS) in the TME strongly correlates with increased clinical response to ICI and favorable patient outcomes in various tumors (10–13).

TLS are ectopic aggregates of organized immune cells formed at sites of chronic inflammation and are seen in cancer, chronic infections, and autoimmune diseases (14, 15). TLS share functional and structural similarities with secondary lymphoid organs (SLOs). However, TLS, unlike SLO, lacks encapsulation and is formed in non-lymphoid tissues (16). SLOs (e.g., lymph nodes) are highly organized immune structures where antigen-presenting cells (APC) interact with specific naïve lymphocytes to initiate adaptive immune responses (17). Activated immune cells then migrate from the lymph nodes to stimulate antitumor reactions at the tumor site. SLOs have been considered the only source of anti-tumor immune response for many years. However, emerging studies have revealed the contribution of TLS to the adaptive immune response (16). The presence of Intratumoral and peritumoral TLS has been reported to increase survival rates in patients with oral cancer, melanoma, pancreatic ductal carcinoma, breast cancer (BC), colorectal carcinoma (CRC), and early hepatocellular carcinoma (HCC) (7, 18–20). Due to the significance of TLS in various cancers, researchers have explored different techniques to induce their development *in vivo* (21). However, current methods have not recapitulated the fully mature TLS clinically associated with a favorable prognosis. Also, TLS produced by these different approaches has been inconsistent, thereby stalling therapy development in this setting (15). This review discusses the current methods to generate TLS and the putative TLS biomarkers. The review further explores epigenetic therapies (epi-therapy) and ongoing clinical trials on epi-therapy in cancers. Finally, the review elucidates the epigenetic regulation underlying TLS formation and highlights potential epigenetic targets to initiate TLS neogenesis *in vivo.*

## 2 Formation of tumor associated tertiary lymphoid structures

**Figure 1:**
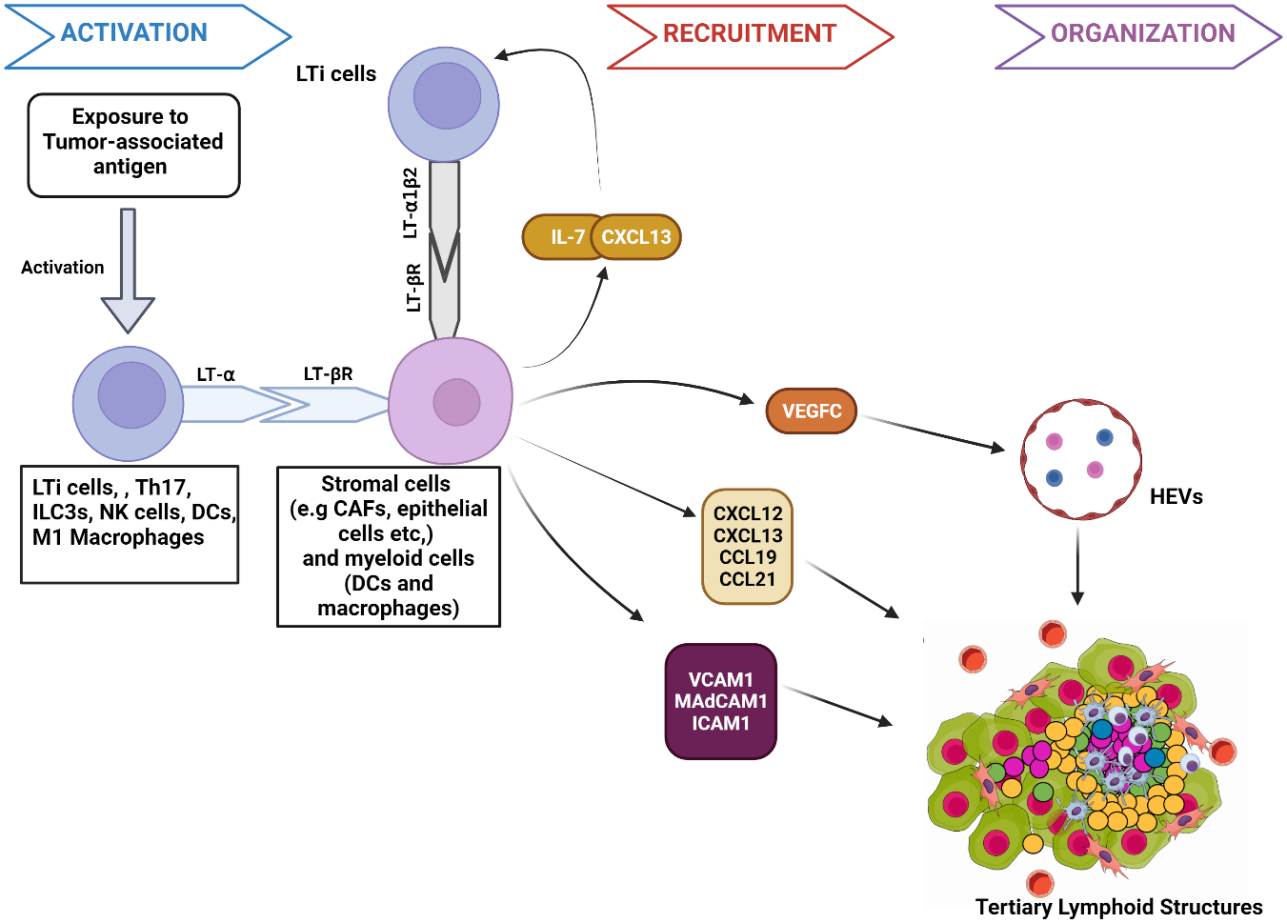
Mechanism of tumor associated tertiary lymphoid structures formation. 1. Activation: The presentation of tumor-associated antigens activates lymphoid tissue-inducer cells (LTi) to express the membrane protein lymphotoxin- α (LT- α). Other immune components, such as M1 macrophages and DC, have the potential to secrete LT- α when activated by specific antigens. Through expressed LT- α, immune components bind to lymphotoxin-β receptor (LT-βR) on stromal cells. The binding releases chemokines such as Interleukin-7 (IL-7) and CXCL13 that interact to facilitate the further immune cell recruitment and binding of LTi cells expressing lymphotoxin- α1β2 (LT- α1β2) to stromal cells. LT-βR binding also stimulates the release of other molecules responsible for the recruitment of immune cells to TLS, such as chemokines (CXCL12, CCL19, and CCL21), Adhesion molecules (VCAM1, MAdCAM1, ICAM1) and VEGFC. 2. Recruitment: VEGFC induces HEV formation in TLS. VCAM1, ICAM1, and MAdCAM1 enhance immune cell trafficking to the TLS. Similarly, CXCL12, CXCL13, CCL19, and CCL21 further signal the recruitment of T-cells and B cells to TLS. 3. Organization: Chemokines facilitate the organization of recruited immune cells into specific zones, such as germinal centers within TLS. Figure Made in BioRender.com.

Studies in preclinical mouse models show that the interactions between stromal cells and hematopoietic lymphoid tissue-inducer cells (LTi) initiate TLS and SLO development (22, 23) (**Figure 1**). TLS development starts when APCs deliver tumor-associated antigens to adaptive immune cells, resulting in lymphocyte activation and cytokine secretion [e.g., Interleukin-7(IL-7)] (24). These activated cells secrete lymphotoxin-α (LT-α) through which they bind to the lymphotoxin-β receptor (LT-βR) expressed on stroma cells (25). This interaction triggers the production of chemokines, including chemokine CXC-chemokine ligand 13 (CXCL13), CXCL12, CC-chemokine ligand 19 (CCL19) and CCL21 (26). CXCL13 and IL-7 interaction recruits LTi expressing lymphotoxin- α1β2 (LT- α1β2), which binds to the LT-βR on tumor stromal cells (22). This binding facilitates the stromal secretion of vascular endothelial growth factor C (VEGFC) and adhesion molecules such as vascular cell adhesion molecule 1 (VCAM1), mucosal addressin cell-adhesion molecule 1 (MAdCAM1), and intercellular adhesion molecule 1 (ICAM1) (27). VEGFC signals the production of high endothelial venules (HEV) responsible for the vascularization of TLS (28). The adhesion molecules and HEV promote further recruitment of immune cells to TLS. The homeostatic chemokines CCL19, CCL21, and CXCL13 stimulate the expression of LT- α1β2 on T-cells and B-cells essential for their segregation into T-cell zones and B-cell germinal centers (GC) respectively in TLS (27).

TA-TLS exhibits different levels of maturity depending on the presence or absence of some structural components, including T-cells, B-cells GC, Follicular Dendritic cells (FDC), or Follicular Reticular cells (FRC), dendritic cells (DC), and HEV (29). Fully mature or classical TLS contains defined GC and T-cell zones interspersed with FRC/FDC-like cells (30). Some non-classical TLS can have dense lymphocytes with no FDC/FRC-like cells and no zonal segregation, while other non-classical TLS contain FDC/FRC-like cells but lack GC reactions. Due to the prognostic advantage of classical TLS, techniques to induce their formation in tumors are being developed (31). Tumor TLS can be located within the tumor (intratumoral) or in the periphery of tumors within 2mm (peritumoral) (18, 32, 33). Although TLS location affects tumor immunogenicity, there is still no observed advantage of intratumoral TLS over peritumoral TLS.

## 3 Induction of TLS: current and potential strategies

**Figure 2:**
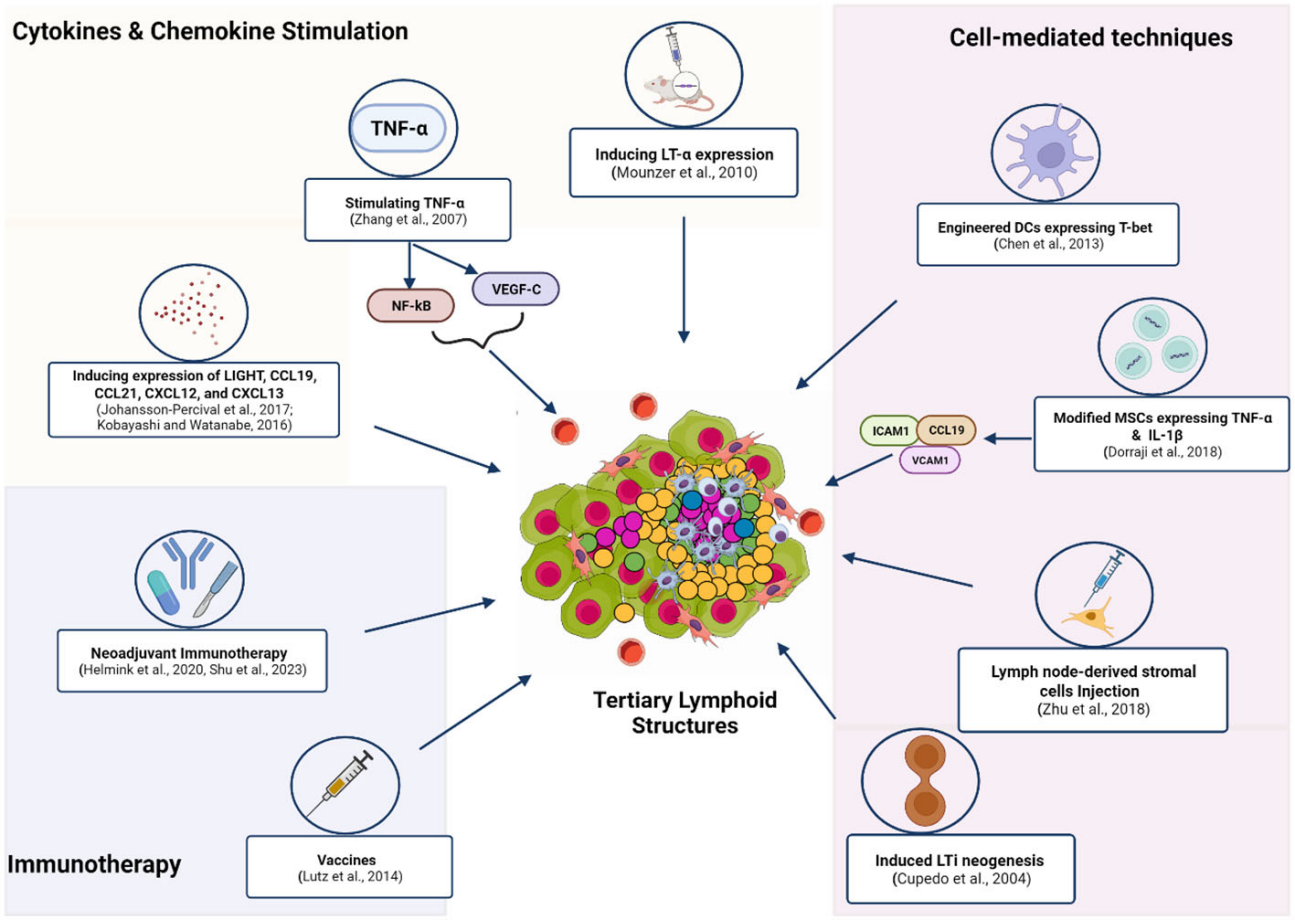
Methods and techniques used to induce the *in vivo* formation of TLS. These strategies employ exogenous compounds to induce cytokines and chemokines to trigger downstream signaling that enhances TLS formation. Neoadjuvant Immunotherapy (ICI and chemotherapy) or (ICI and surgical resection) promotes TA-TLS neogenesis. Administration of the allogeneic vaccine (GVAX) for Pancreatic ductal adenocarcinoma (PDAC) induced TA-TLS formation. Immune cell-induced expression of certain TF or chemokines was applied to form TLS at tumor sites. Increasing the number of LTis available for activation by APC was also employed by another study to stimulate TLS genesis. Figure Made in BioRender.com.

Given the association of TLS with a favorable response to immunotherapy, studies have employed various approaches to induce TLS formation *in vivo*. Local induction of TLS in murine models has been achieved by stimulating the expression of chemokines and cytokines such as LT- α (34), TNF- α (35), LIGHT (36), CXCL13 (37), CCL21, CCL19, and CXCL12 (38). (**Figure 2** **).** In these studies, cytokine/chemokine-induced TLS led to better antitumor responses (39–41). Interestingly, some studies have reported increased TA-TLS in patients receiving immunotherapy. For example, a phase 2 study of melanoma patients taking ICI reported increased B-cell activity and TLS formation, which correlated with a positive response (11). This study indicated the potential of ICI to stimulate TLS induction. Similarly, the administration of neoadjuvant immunotherapy (ICI before surgical resection) in HCC produced varied TLS, suggesting that TLS induction might be favored by neoadjuvant therapy. Moreover, TLS neogenesis observed in this study corresponded with an increased expression of memory T-cell markers. These findings highlight the possible contribution of TLS in the physiological process of memory cell generation after immunotherapy (42). Another study in non-small cell lung cancer (NSCLC) patients receiving neoadjuvant immunotherapy (ICI and chemotherapy) found higher TLS abundance and maturation in patient tissues. Increased levels of TLS in this study correlated with more prolonged disease-free survival (43). Association between the administration of neoadjuvant nivolumab and the formation of TLS with high B-cell activity was also reported in a trial involving melanoma patients (11, 44). These studies uncover a relationship between neoadjuvant therapy and TLS; however, the directionality or precise mechanisms of these associations are still uncharacterized (45). Other immunotherapy approaches, such as the administration of an allogeneic vaccine (GVAX) for pancreatic ductal adenocarcinoma (PDAC), have been shown to induce intratumoral TLS in 85% of the vaccinated patients (46). Cell-mediated strategies to stimulate TLS neogenesis have been tested. Some of the cell-mediated approaches to form TLS include ectopic injection of LTi cells, intratumoral injection of DC expressing T-box transcription factor TBX21 (T-bet), and the induced expression of TNF- α and IL-1β by mesenchymal stem cells (MSC) (47–49) (**Figure 2**).

Despite the apparent proliferation of strategies to form TLS, the multi-step complexity of their induction and the heterogeneity of TA-TLS have stunted the growth in this area of research (11, 15). Also, existing methods are focused mainly on systemic cytokine and chemokine stimulation, which poses a dilution effect risk and the possibility of systemic cytokine dispersion. In animal studies, these events have triggered significant toxicity and tissue damage (50, 51). Hence, there is a need to explore novel techniques to stimulate the local or targeted production of these proinflammatory molecules. In addition, understanding the molecular control behind TLS formation could help identify targets for genetic or epigenetic therapy to induce site-specific TLS (24). Below, we discuss the potential of viral-mediated targeted delivery of chemokines for local stimulation. We also highlight the prospects of epigenetic therapy and identify novel epigenetic targets for the molecular stimulation of TA-TLS.

### 3.1 Viral vector-mediated tissue-specific delivery of chemokines and cytokines

Local chemokine production at tumor sites could circumvent toxicity derived from ectopic expression. Viral vector-mediated delivery or adoptive transfer of genetically modified cells has been shown to enhance the expression of molecules at the anatomical target site. Many studies employ viral vectors carrying cytokines and gene-modified immune cells to induce TLS in preclinical and early-stage clinical studies (52). Critical considerations of a viral vector-based strategy include the selection of the optimal cell in the TME that can be targeted to generate stable and functional TLS at the tumor site. Another important consideration is choosing a safe viral vector system to efficiently deliver transgene to target cells.

Adoptive transfer of TIL engineered to express IL-2 has shown significant efficacy in clinical trials for patients with multiple myeloma (53, 54). Most T-cells within TILs are known to be non-tumor specific, potentially leading to a decreased or null retention of transgene-expressing T-cells at tumor sites (55). Furthermore, intratumoral injection of DC expressing CCL21 transduced with adenoviral vectors (AAV) increased CD8 infiltration and enhanced antitumor responses in an NSCLC phase I/II clinical trial (36). Also, CAR-T cells, known for their value in hematological cancer treatment, constitute a promising tool for engineering the TME (56, 57). CAR-T cells expressing CCL19 and IL-7 induced endogenous T-cell recruitment to tumors, shifting the immunosuppressive phenotype of the TME (58). Another exciting study employed engineered T cells expressing the proinflammatory toll-like receptor-5 (TLR-5) agonist, flagellin, to modify the immunogenic “cold” tumor phenotype (59). The safety of CAR-T cells was improved by masking the antigen-binding site via a protease-sensitive linker, which becomes activated upon encountering tumor proteases. This specialized activation helps to reduce “on-target off-tumor” toxicities (60). About vector systems, AAVs are currently the most utilized vector tools for stable gene transfer; however, their efficiency is limited by the presence of circulating neutralizing antibodies (61–63). In contrast, lentiviral vectors (LV), which are largely HIV-based, have transduction efficiencies similar to AAV but with no preexisting immunity (61). In addition, LVs are efficient vehicles for introducing and stably expressing effector molecules in healthy human cells, tumor cells, and immune and non-immune cells (57, 64–66). Moreover, LVs have extensive plasticity to accommodate several genes and regulatory sequences (52). For example, LVs have been used to deliver engineered CAR-T cells expressing CCL19 and IL-7 to promote TME infiltration and enhance antitumor response (67–69). Another advantage of LVs is their ability to deliver the CRISPR/Cas9 systems due to their efficient capacity to transport across the nuclear membrane in resting cells (70). This transport capacity opens up enormous possibilities for site-specific editing of target genes and regulatory sequences essential to TLS formation. The combination of vector and genome editing techniques offers excellent prospects for *in vivo* TLS induction; however, this potential has not yet been demonstrated.

### 3.2 Epigenetic modulation in cancer therapy

Using epigenetic inhibitors to modulate the expression of critical tumor-associated genes represents one of the novel approaches in cancer therapy. Epigenetic mechanisms are inheritable changes that affect gene expression without altering the DNA sequence. Many studies have linked modifications in the epigenome with the occurrence, progression, and resistance of many tumors (71, 72). Furthermore, epigenetic alterations in the TME have been linked to the formation of an immunosuppressive TME (73). Hence, researchers are exploring tools to manipulate the TME epigenome to enhance immunotherapy response (74). Epigenetic mechanisms that regulate gene activity include DNA methylation, histone modification (acetylation, methylation, phosphorylation, etc.), and non-coding RNAs. These alterations in the epigenome are controlled by three groups of proteins (or enzymes). Writers- stimulate the addition of the epigenetic groups on the DNA or histone tail. Examples of writer proteins are DNA methyltransferases (DNMT), histone acetyltransferases (HAT), histone methyl transferases (HMT), and ubiquitin E3 ligases.

Furthermore, reader proteins (e.g., bromodomain and chromodomain) are involved in recognizing modified histones and recruiting epigenetic marks. Finally, erasers catalyze the removal of epigenetic marks on modified DNA or histones (75–78). Epigenetic erasers include Ten-eleven translocation (TET) enzymes, histone deacetylases (HDAC), histone demethylases (HDM), and deubiquitinating enzymes. The writers, readers, and erasers represent the workforce behind epigenetic changes that activate or silence tumor-associated genes (78). This is why most translational interventions to manipulate the epigenome in cancer have primarily targeted these functional proteins.

#### 3.2.1 DNA methylation in cancer, TME, and therapy

Methylation pattern changes were the first identified epigenetic alteration in cancer. Hypermethylation at the promoter region of the tumor suppressor gene (TSG) blocks the binding of transcription factors, thereby preventing transcription activation. Inhibiting transcription of TSG contains their expression, leading to cancer growth and progression (79). For example, Promoter hypermethylation repressed BRCA1 expression, resulting in breast cancer growth and metastasis (80, 81). On the other hand, hypomethylation at the promoter region causes overexpression of genes (82). Hypomethylation at the promoter region of oncogenes and proto-oncogenes can stimulate cancer development and metastasis. Promoter Hypomethylation in the long interspersed nuclear element-1 (LINE-1) gene was found to promote colorectal cancer metastasis (83).

The covalent transfer of methyl group to the fifth carbon of cytosine (5mC) is mediated by the members of the DNMT enzyme family (DNMT1 (most common), DNMT2, DNMT3a, and DNMT3b). In contrast, demethylation is facilitated by the TET hydroxylase enzymes. TET-mediated demethylation involves the conversion of 5mC to multiple groups such as 5-hydroxymethylcytosine (5hmC), 5-formylcytosine (5 fC), and 5-carboxylcytosine (5caC) and unmethylated cytosine (75, 79).

In an extensive study by Liu et al., modifications in the 5mC signature were observed to mediate TME remodeling in lung cancer tissues. Furthermore, the study found that patients with low methylation levels had higher levels of TIL and enhanced clinical response (84). In another study, the methylation of the promoter region of the Egfl7 gene within the tumor stroma inhibited the expression of CCL2. CCL2 suppression played a role in fostering an immunosuppressive TME and promoting breast cancer metastasis (85). More studies have identified links between cancer resistance development and DNA methylation alterations. These findings imply a promising potential for using methylation modifiers to enhance levels of TIL and bolster the response to immunotherapy (73, 86).

The Food and Drug Administration (FDA) has approved seven epigenetic drugs (epi-drugs) for treating cancer. Among these medications, six are designated for treating blood-related cancers, whereas only one holds approval for solid tumors. Within this group, two DNMT inhibitors—5-azacytidine and 5-aza-2-deoxycytidine (or Decitabine)—are approved for treating myelodysplastic syndrome (MDS). These drugs have demonstrated efficacy by inhibiting DNMT, thereby reversing abnormal hypermethylation of tumor suppressor genes (TSGs), leading to TSG reactivation, halting proliferation, and inducing cell death (87). DNMT inhibitors are used in cancer treatment with either immunotherapy or chemotherapy agents. Furthermore, many clinical trials still employ DNMT inhibitors to potentiate therapy response in refractory tumors (79, 88).

#### 3.2.2 Histone modification in cancer, TME, and therapy

The N-terminal segments of histone proteins, abundant in lysine and arginine, serve as crucial sites for frequent modifications by epigenetic writers. Post-translational alterations in histones control the accessibility and structure of chromatin, impacting gene expression during transcription (89). Common modifications in histones are acetylation, deacetylation, methylation, and phosphorylation. Other recognized histone changes include ubiquitination, citrullination, formylation, deamination, butyrylation, etc.

##### 3.2.2.1 Acetylation and deacetylation

Enzymes, Histone acetyltransferase (HAT), and HDAC catalyze histone acetylation and deacetylation, respectively. HAT adds acetyl groups (-CH3CO) to lysine residues, neutralizing the positive charge of histones. The neutralization initiates chromatin transition to a relaxed state (euchromatin), facilitating transcription activation by enabling TF binding (75, 89). Conversely, HDAC removes acetyl groups, leading to chromatin condensation into a heterochromatin state that impedes TF access. The acetylation on histone H4 at lysine 16 (H4K16Ac) and 122 (H4K122Ac) orchestrates shifts in chromatin structure, influencing transcription activation (90, 91). The balance between HAT and HDAC expression regulates chromatin structure and gene expression. Disruption of this balance may trigger oncogenic signaling, leading to cancer growth. For example, over-expression of HDAC enhanced tumor progression in triple-negative breast cancer (TNBC) patients, whereas decreased HDAC levels have been shown to cause metastasis in cervical cancer (92, 93).

A comprehensive study by Xu et al. unveiled a correlation between histone acetylation (HA) and immunosuppressive signaling within HCC samples. Patients with low HA scores displayed enhanced responsiveness to immunotherapy compared to those with a high HA score (94). Given the significance of histone acetylation and deacetylation in cancer progression and resistance, epigenetic drugs have been developed targeting acetylation levels for certain malignancies. Currently, four FDA-approved HDAC inhibitors are in clinical use. The first and second HDAC inhibitors, suberoylanilide hydroxamic acid (SAHA, Vorinostat) and romidepsin, respectively, are approved for treating cutaneous T-cell lymphoma (CTCL). Belinostat, the third approved HDAC inhibitor, is used in peripheral T cell lymphoma (PTCL) treatment, while Panobinostat was the fourth HDAC inhibitor approved for treating multiple myeloma. Blocking HDAC activity accumulates acetylated histones and proteins, triggering cell cycle arrest and cell death (95–97). These drugs are designed based on the knowledge that hematological malignancies are the most amenable to epigenetic alterations. Ongoing clinical trials are exploring the effectiveness of these drugs in treating solid tumors.

##### 3.2.2.2 Methylation

Histone methyltransferases (HMT) regulate the methylation of arginine and lysine residues at H3 and H4 positions on the histone tail. On the other hand, histone demethylases (HDM) catalyze the removal of methyl groups from these residues. The methylation’s resultant effect could be transcription activation or repression, depending on the methylated residue. For instance, trimethylation of lysine 4 on H3 (H3K4me3) leads to transcription activation, while trimethylation of lysine 9 (H3K9me3) and 27 (H3K27me3) on H3 resulted in transcriptional repression (98–100). Enhancer of zeste homolog 2 (EZH2) is an enzymatic catalytic subunit of polycomb repressive complex 2 (PRC2) that catalyzes the trimethylation at lysine 27 on H3 (H3K27me3) (101).

EZH2-mediated trimethylation was found to alter the downstream expression of some TSG. As a result, EZH2 stands as an essential therapeutic target in various cancers, prompting the testing of multiple EZH2 inhibitors in clinical or preclinical studies (102). Changes in the histone methylation patterns have been linked to TME modulation by several studies. A recent study by Yu et al. showed that changes in methylation pattern on histone H4 were essential for TME heterogeneity in HCC samples (103). Another study observed that EZH2-induced epigenetic silencing of miR-29b/miR-30d inhibited macrophage infiltration into the TME, thereby augmenting breast cancer progression. In addition, EZH2 inhibition reduced tumor burden and slowed progression in breast cancer both *in vivo* and *in vitro* (104). Furthermore, an auto-regulatory feedback loop involving EZH2, HOTAIR, and miR-193a was found to promote prostate carcinogenesis and metastasis (105).

Due to the widespread tumorigenic role of EZH2 and its overexpression in various cancers, EZH2 inhibitors are being evaluated in many clinical and preclinical studies. Tazemetostat is the only FDA-approved EZH2 inhibitor for treating advanced epithelioid sarcoma (106). Research into tazemetostat has shown its sufficient capacity to demethylate H3K27me3 and upregulate the expression of the chemokine CCL17 in B-cell lymphoma cells. Additionally, Tazemetostat promoted T-cell recruitment, indicating potential use in combination with immunotherapy treatment (107). Ongoing clinical trials are investigating the efficacy of EZH2 inhibitors either as a monotherapy or in combination with immunotherapy for treating malignancies. Beyond EZH2 inhibitors, other drugs targeting HMTs and HDMs are currently evaluated in clinical trials. For example, tranylcypromine (TCP), an FDA-approved drug for depression and anxiety, is being repurposed in clinical studies as a lysine demethylase (LSD) inhibitor. Combined with all-trans-retinoic acid (ATRA), TCP is being explored for acute myeloid leukemia treatment (108).

#### 3.2.3 Noncoding RNA in cancer, TME, and therapy

Non-coding RNAs (ncRNA) comprise approximately 98% of the human genome. Although there is still much unknown about ncRNA, their capacity to modulate the expression of various tumor-associated genes and regulate pathways linked to cancer proliferation, metastasis, and resistance has been established. ncRNA are classified based on their length into small (sncRNA) and long non-coding RNAs (lncRNA, more than 200 nucleotides) (109). The sncRNA is involved in RNA interference (RNAi), forming complexes with other proteins to target and silence complementary mRNA transcripts. The sncRNA consists of microRNAs (miRNA), PIWI-interacting RNA (piRNA), endogenous small-interfering RNA (endo-siRNA), and small nucleolar RNAs (snoRNA). Their highly conserved nature spans across different species.

On the other hand, lncRNA lacks conservation among species, including long enhancer ncRNAs, long intergenic non-coding RNAs (lincRNAs), and transcribed ultraconserved regions (T-UCR). T-UCRs are conserved in humans, mice, and rats (110, 111). Dysregulation in both lncRNA and sncRNA is observed across various cancer types. For example, HOX antisense intergenic RNA (HOTAIR) interacts with PRC2 to induce methylation and silencing of TSG in endocrine and prostate cancer (105, 112). Altered expression of maternally expressed 3 (MEG3), a tumor-suppressing lncRNA, was observed to enhance epithelial-to-mesenchymal transition (EMT) in several solid tumors (113, 114). Other lncRNAs contributing to tumor proliferation and immunosuppressive signaling in the tumor microenvironment include BRAFP1, NANOG, and MALAT1 (115). SncRNAs, particularly miRNAs, are established regulators of tumor growth and metastasis. Some miRNAs, like miR-15/16, miR-29, and miR-34, exhibit tumor-suppressive functions, while miR-21, miR-155, and miR-221/222 act as oncogenes (116). Overexpression of oncogenic miRNA (oncoMIR) and downregulation of tumor suppressive miRNA are seen in various solid and hematological malignancies (109, 117). Efforts to inhibit overexpressed ncRNAs involve ongoing investigations using antisense anti-oligonucleotides (ASOs), antagomirs, siRNAs, and short hairpin RNAs (shRNAs) in both *in vitro* and *in vivo* studies. CRISPR/Cas9-based genome editing has also shown promise inhibiting ncRNA expression (118). Although there are FDA-approved ASO and siRNA, such as Fomivirsen and Patisiran, for other diseases, no approved ncRNA inhibitor exists for cancer treatment.

Nevertheless, various preclinical and clinical studies are currently exploring the therapeutic potential of lncRNA inhibitors in cancer (119, 120). In contrast to inhibiting ncRNAs, different strategies to restore normal expression levels of some downregulated tumor suppressive ncRNA are being explored. Synthetic ncRNA-like molecules, such as miRNA mimics, are currently employed to restore the expression of downregulated ncRNA *in vivo*. However, a better understanding of these synthetic molecules is essential before their application in clinical trials.

#### 3.2.4 Epigenetic drugs in ongoing clinical trials

Numerous clinical studies explore epigenetic modifiers as adjunct therapies for various cancers. Epigenetic drugs (Epi-drugs) belonging to different classes are currently being evaluated in multiple studies either as a monotherapy or in combination with immunotherapy. Many ongoing trials focus on investigating the enhanced antitumor activity achieved by combining the DNMT inhibitor, Decitabine, with radiation, immunotherapy, and chemotherapy drugs across multiple malignancies (NCT05178693, NCT02159820, NCT05089370, NCT03417427, NCT03240211) (**Table 1**). Other studies evaluate the efficacy of the second FDA-approved DNMT inhibitor, 5-azacytidine, when combined with chemotherapeutic agents in managing AML (NCT04248595). Several trials delved into the potential therapeutic benefit of combining two epigenetic modifiers. For instance, the safety and the antitumor response from administering 5-azacytidine and Decitabine are being assessed in patients with myeloid malignancies (NCT04187703).

**Table 1:** Recruiting clinical trials employing epigenetic therapy.

Trial Number	Epigenetic Class	Epigenetic Drug	Regimen	Mechanism & Disease	Status
NCT05178693	DNMT Inhibitor	Decitabine	Combination with Cedazuridine+ Lutathera (Radiation)	Epigenetic modification of Somatostatin Receptor-2 to improve treatment outcome in Metastatic Neuroendocrine Tumours	Recruiting
NCT02159820	DNMT Inhibitor	Decitabine (Low Dose)	Combination with Carboplatin-Paclitaxel	To induce epigenetic reprogramming of tumor cells and immune cells to improve response to Carboplatin-Paclitaxel in Advanced Ovarian Cancer treatment	‘‘
NCT03366116	DNMT Inhibitor	5-aza-4’-thio-2’-deoxycytidine (Aza-TdC)	Monotherapy	To establish the safety and tolerability of Aza-TdC in patients with advanced solid tumors	“
NCT04187703	DNMT Inhibitor	5-azacitidine plus decitabine	Combination of 5-azacitidine and decitabine (5AZA-alt-DEC)	To test the efficacy and safety of the combination (5AZA-alt-DEC) in patients with myeloid malignancies	“
NCT05089370	DNMT Inhibitor	Decitabine	Combination with Cedazuridine+ Nivolumab	To enhance the efficacy of Nivolumab by inducing epigenetic modulation in patients with metastatic mucosal melanoma.	“
NCT03417427	DNMT Inhibitor	Decitabine	Combination with Cytarabine	To assess the increased efficacy of combining demethylating agents with chemotherapy in intermediate-risk AML	“
NCT03240211	DNMT Inhibitor	Decitabine	Combination with Pembrolizumab	To induce immunomodulation by epigenetic therapy and enhance anti-tumor activity in patients with PTCL and CTCL.	“
NCT04248595	DNMT Inhibitor	5-azacitidine	Combination with homoharringtonie-based regimen (AZA-HHT).	To assess the safety and efficacy of AZA-HHT combination in the treatment of AML	“
NCT03999723	DNMT Inhibitor	5-azacitidine	In combination with Oral Vitamin C, 1000mg	To test the therapeutic potential of combining an active hypomethylating agent with a passive hypomethylating agent in patients with high-risk MDS and chronic myelomonocytic leukemia (CMML)	“
NCT04407741	EZH2 Inhibitor	SHR2554	Combination with anti-PD-L1/TGFβ antibody, SHR1701	To assess the epigenetic modulating capacity of SHR2554 and the efficacy of its combination with SHR1701 in patients with metastatic solid tumors and refractory B-cell lymphomas	“
NCT04705818	EZH2 Inhibitor	Tazemetostat	Combination with Durvalumab	To enhance the efficacy of durvalumab in the treatment of pancreatic, colorectal cancer and other types of solid tumors.	“
NCT05317403	DNMT Inhibitor plus HDAC Inhibitor	5-azacitidine plus vorinostat	Combination with venetoclax and chemotherapy drugs	To enhance the therapeutic outcome of young patients with relapsed acute myeloid leukemia (AML)	“
NCT05573035	-	LYL845, an epigenetically reprogrammed TIL	Monotherapy	To assess the safety and the anti-tumor activity of LYL845 in metastatic melanoma, NSCLC, and CRC	“
NCT05873244	HDAC Inhibitor (selective)	Zabadinostat (CXD101)	Combination with the anti-PD-1 medication, geptanolimab	To induce epigenetic modifications that enhance susceptibility to immunotherapy in resistant HCC patients.	“
NCT05400993	HDAC Inhibitor (selective)	Chidamide	Combination with Chemotherapy drugs (anthracycline plus paclitaxel)	To test the anti-tumor efficacy of Chidamide with Chemotherapy as a Neoadjuvant treatment of HR-positive/HER2-negative BC.	“
NCT03903458	HDAC Inhibitor	Tinostamustine	Combination with Nivolumab	To assess the safety and tolerance of the combined therapy in end-stage metastatic melanoma patients.	“

Similarly, another clinical study measures the combined activity of 5-azacytidine and a passive hypomethylating agent (Vitamin C) (NCT03999723). HDAC inhibitors are also being evaluated in various clinical studies. Selective HDAC inhibitors like Chidamide and Zabadinostat are being used in trials in combination with other anticancer drugs for treating HR-positive/HER2-negative BC and refractory HCC, respectively (NCT05873244, NCT05400993).

The clinical efficacy of combining vorinostat with 5-azacytidine and other chemotherapy drugs is also being evaluated in an ongoing trial for patients with relapsed AML (NCT05317403). Epigenetic modulating capacity and the anti-tumor response of administering EZH2 inhibitors, SHR2554, with an anti-PD-L1 antibody, SHR1701, is being tested in a trial involving patients with metastatic solid tumors and resistant B-cell lymphomas (NCT04407741). The safety and therapeutic potential of administering epigenetically reprogrammed TIL, LYL845, is extensively studied in a trial involving metastatic melanoma, NSCLC, and CRC patients. The vast number of ongoing clinical trials underline the promise of epigenetic therapy (epi-therapy) in cancer management. Although they are used primarily with other anticancer drugs, epi-drugs have shown promise in enhancing antitumor responses and reversing resistant phenotypes. The future of cancer treatment seems poised to incorporate epi-therapy to modulate the immunosuppressive TME and augment TSG expression. However, there is still a selectivity problem and a need to improve the epi-therapy targeting to bolster immune infiltration and antitumor response (88, 121).

### 3.3 CRISPR-based epigenetic editing

The lack of specificity of epi-drugs and reported off-target effects have prompted the exploration of alternative methods for *in vivo* epigenome modulation. The need for alternatives birthed the idea of utilizing CRISPR-mediated technology to induce epigenome editing (122). CRISPR-based epigenome editing employs dead or inactivated Cas9 protein (dCas9), a mutated version of Cas9 with comparable DNA binding affinity but lacks the nucleolytic activity of normal Cas9 protein. In the CRISPR-dCas9 system, epigenetic modifying domains are fused with the dCas9 protein. Then, in association with guide RNA, the complex targets the specific genetic locus where the modifying domain performs the editing functions. Various modifying or effector proteins can be fused to dCas9 to initiate specific epigenetic modifications. Epigenetic modifiers (EM) such as DNMT3A or TET1 protein can be fused to dCas9 to induce gene-specific methylation or demethylation, respectively (122, 123). Similarly, transcription modifying proteins (TMP) can be complexed with dCas9 to initiate specific transcription activation or repression. There is great potential for CRISPR-dCas9 application in modulating the epigenome to enhance therapeutic outcomes in patients with various tumors. However, there are limitations to the clinical applications of CRISPR-based delivery systems. Even though the CRISPR-dCas9 system helps to improve the precision and specificity of gene editing, reported cases of off-target effects restrict their widespread application in clinical studies. Methods to mitigate these off-target effects have been developed, which could potentially advance the clinical use of CRISPR systems (122, 124, 125). Another limitation of using CRISPR-based epigenetic editing is the large size of CRISPR-Cas9 systems. This limitation restricts the types of EM or TMPs fused with Cas9 systems.

### 3.4 Epigenetic regulation of TA-TLS neogenesis

Despite considerable research in epigenetic therapy, methods to stimulate epigenetic-mediated immunomodulation are still unclear. Also, epi-drugs have shown more therapeutic benefits in hematological cancers than solid tumors. This primarily stems from genomic complexity, reduced drug/immune infiltration, and intratumoral heterogeneity in solid tumors. It is then imperative to explore novel epigenetic strategies to enhance immune infiltration and mitigate the burden of solids. Given that the presence of TLS in most solid tumors correlates with improved immune activity and therapeutic success, uncovering the epigenetic regulations underlying TLS neogenesis could unveil new epigenetic targets to induce TLS formation across various tumors. Below, we will delve into the epigenetic regulations underlying the formation of essential molecules and interactions necessary for TLS neogenesis.

#### 3.4.1 Innate lymphoid cells

The activation of CD4^+^ CD3^-^ CD45^+^ innate lymphoid cells (LTi) by antigen exposure signals the early processes of SLO or TLS formation (15). LTi differentiate from fetal liver precursors, and they express the RORγt and Id2 transcription factors (TF) (126). LTi shares a similar origin with Innate lymphoid cell 1 (ILC1), ILC2, and ILC3. Lineage-specific TFs control the final differentiation process into each lymphoid cell type. *In vitro* studies with fetal transitional Innate Lymphoid Cell Precursor (ftILCP)-cells with Lin^−^IL7Rα^+^α_4_β_7_^+^Id2^+^ phenotype- indicated that TFs such as T-bet, RORγt, and Gata3 control the differentiation into the three ILCs and LTi (127–129). Epigenetic changes and reprogramming guide the expression of these TF (130). For example, T-bet induces transcription activation during specific lineage differentiation by chromatin remodeling and histone modification (131). T-bet interacts with H3K27-demethylase and H3K4-methyltransferase activities to perform these functions (132, 133). Gata3 controls lineage differentiation through histone acetylation at the H3K14 locus and methylation at H3K4 (134). Another precursor for the innate lymphoid cells is the common helper innate lymphoid progenitor (CHILP) (126). The negative transcription regulator Id2 is essential for differentiating CHILP from LTi (127). Although the epigenetic mechanisms behind Id2 immune differentiation function are unclear, changes in methylation patterns and histone deacetylation have been shown to control Id2 and Id4 expression during oligodendrocyte differentiation (135, 136). TFs such as Tcf7, Nfil3, and Tox function by stimulating epigenetic reprogramming, and they are critical for forming the three ILCs and LTi from early innate lymphoid precursors (EILP) (26, 130). ILC3 and Th17 share the expression of retinoic acid-related orphan receptor γt (RORγt) with LTi and can act as surrogates for LTi (14). Other immune cells, such as Natural Killer (NK), B-cells, CD8^+^ T-cells, and macrophages, can also be potential surrogates for LTi (31, 137, 138). The presence of multiple surrogates of LTi offers the potential to modify existing immune cells of the TME to form LTi cells or express LT- α

#### 3.4.2 Lymphotoxin-αβ

LT-α and LT-β, membrane-bound proteins expressed on activated immune cells, are also critical in generating TLS. LT-α (formerly known as TNF-β) and LT-β are members of the tumor necrosis factor (TNF) family, sharing similar signaling pathways and receptors with other family members (139). LT-α proteins are expressed on activated immune components, while their receptors (e.g., LT-βR) are expressed on stromal or epithelial cells. LT-α and LT-β can differentiate into membrane-bound heterotrimers, LT-α_1_β_2_ and LT-α_2_β_1_, who can bind to LT-βR to stimulate lymphotoxin signaling (140). It was observed that the expression of LT-α on immune cells was independent of cytokine and chemokine signaling, thereby proposing that a different mechanism underlies LT-α expression (141). Although the mechanism is not yet unraveled, studies have found that the expression of cytokines of the TNF family is primarily regulated by epigenetic modulation. For example, extensive demethylation was observed at the TNF-α locus of cells that rapidly express TNF-α (142). Furthermore, histone acetylation (H3 and H4) at the promoter (TNF4) and enhancer regions of TNF-α controls the expression of TNF-α (142). Since LT- α is a member of the TNF family, these findings support the idea that epigenetic reprogramming could also stimulate its expression. Further research into the epigenetic control of LT-α expression by immune cells is required to identify potential targets.

#### 3.4.3 Ligand binding to LT-βR

LT-α, through its heterotrimer LT-α_1_β_2,_ binds to LT-βR receptors to initiate the downstream release of chemokines, cytokines, and adhesion molecules essential for forming TLS. LT-βR receptors are transmembrane proteins expressed by stromal cells [e.g., fibroblasts, epithelial cells, and endothelial cells (EC)] and myeloid cells (e.g., DC and macrophages) in the TME. LT-βR binds to two ligands, LTαβ and lymphotoxin-like cells (LIGHT) (140, 143). Ligand attaching to LT-βR activates signaling of the nuclear factor κB (NFκB) and c-Jun N-terminal kinase (JNK) pathways (144). There are two methods of NFκB activation after LT-βR binding called classical and non-classical NFκB signaling pathways (26). The classical path occurs within minutes and is initiated by IL-1R and TNFR1 proteins. The classical NFκB activation pathway does not require novel gene expression and the recruitment of TRAF-2 proteins (145). In the normal state, the Inhibitor of κB (IκB) maintains NFκB signaling in an inactive state by sequestering the transcription factor RelA/p50 complex in the cytoplasm. During classical signal activation, phosphorylation and ubiquitin-dependent degradation of IκB occurs, liberating RelA/p50 from its complex (146). This allows the nuclear translocation of RelA/p50 to stimulate the release of pro-inflammatory cytokines and adhesion molecules. In contrast, the non-classical NFκB pathways largely depend on the overexpression of NF-Kappa-B-inducing kinase (NIK) protein (145, 146). NIK proteins share similarities with Mitogen-activated protein-3-kinase (MAP3K) and interact with TRAF-2 proteins to stimulate the downstream release of homeostatic chemokines (147). Non-classical signaling constitutes the major LT-βR-induced pathway for releasing chemokines such as CCL19, CCL21, CXCL3, CXCL12, CXCL13, and BAFF. CCL19, CCL21, and CXCL13 are essential for forming SLOs and TLS. Activation across the canonical pathway is also required to produce adhesion molecules- VCAM1, MAdCAM1, and ICAM1- responsible for recruiting immune cells to the TLS (26, 146).

Increasing evidence has shown that the expression of essential components for NFκB activation is controlled by epigenetic remodeling. The expression of TRAF2 was found to be regulated by reduced expression of histone methyltransferase EZH2 (148). This underlying epigenetic mechanism provides an avenue to activate NFκB pathways to form TLS, which can be otherwise inhibited to suppress inflammation in autoimmune diseases. Furthermore, NIK expression and control of non-classical NFκB pathways are regulated by histone acetylation (H3K9) (149). Although further studies into the epigenetic control of NFκB signaling are required, there is a strong potential for regulating this signaling pathway to produce molecules essential for TLS formation.

#### 3.4.4 Homeostatic chemokines

Local stimulation of chemokines- CCL19, CCL21, CXCL12, and CXCL13 have induced TLS in animal models. Although the TLS type produced by each chemokine differed, their ability to form TLS highlights novel mechanisms independent of LTi and LT-βR binding (41). An excellent strategy could involve manipulating cells of the TME to secrete these chemokines. CCL19 and CCL21 are potent leucocyte chemoattractants and orchestrate T-cell and B-cell recruitment into TLS (150, 151). CCL19 can be produced by the stromal cells of lymph nodes, mature DC, the spleen, and HEV (150). Methylation at the promoter region of CCL19 was found to lower its expression in cancer (152). Furthermore, TFs such as STAT and IRF require histone acetylation and chromatin remodeling to regulate the expression of CCL19 (152, 153).

CXCL13 is another potent chemoattractant that facilitates the recruitment of B-cells and the formation of B-cells GC in the TLS. CXCL13 exerts a weak attraction on T-cells and macrophages and stimulates HEV formation in TLS. Cells in the TME have displayed the ability to produce CXCL13. For example, in Angioimmunoblastic T-cell lymphoma, CXCL13 was found to be expressed mainly by FDC (154). Tumor-associated fibroblasts had CXCL13 in response to hypoxia and tissue injury (155). CD8^+^ T-cells and CD20^+^ B-cells were found to express CXCL13 in ovarian cancer (156). Other stromal and EC of the TME have also been found to produce CXCL13 (157, 158). There have been contrasting efforts to use CXCL13 for therapy. Anti-CXCL13 antibodies have been used to stop tumor growth in breast cancer (BC) (159). Alternatively, inducing CD4^+^ expression of CXCL13 allowed the formation of TLS and improved the leucocyte infiltration in ovarian cancer (160). The contrast in strategies indicates that CXCL13 induction to give TLS should be tissue and disease-specific. To ensure this specificity, the knowledge of the molecular underpinnings of CXCL13 expression is necessary. With that said, there is little understanding of the epigenetic regulation of CXCL13 expression. A study of the CXC chemokine family identified that their expression is independent of DNA methylation. However, CXCL8 and CXCR1/2 expression are affected by chromatin remodeling and histone acetylation (161).

#### 3.4.5 Adhesion molecules

Ligand binding to LT-βR also triggers TNF-α signaling and the release of membrane-bound integrin ligands (VCAM1, MAdCAM1, ICAM1). These molecules facilitate leucocyte adhesion and trafficking to the TLS. The EC primarily expresses them, but fibroblasts, keratinocytes, and leucocytes can also express ICAM1 (162). Although these adhesion molecules contribute to TA-TLS formation, research into their therapeutic potential has been conflicting and controversial (163). For instance, ICAM1 expression was associated with higher responsiveness to ICIs and prolonged patient survival (164). Alternatively, over-expression of ICAM1 was associated with tumor metastasis (165). Targeted increases in VCAM1 and MAdCAM1 expression have been considered a potential strategy to manage brain and colon tumor metastasis, respectively (166, 167). However, high expression of VCAM1 correlated with poor prognosis and increased tumor invasion in colorectal cancer (CRC) (168, 169). The double-edged functions of these molecules limit their applicability for therapy. However, understanding the molecular mechanisms regulating the expression of these molecules can enhance their potential as therapy targets. ICAM1 expression is predominantly controlled through transcription. ICAM1 has a specific binding site for NFκB and TNF-α nuclear factor (C/EBP), which, when bound, stimulates its transcription (170). Other pro-inflammatory cytokines, such as IFN-gamma and IL-6, can also trigger ICAM1 expression in cells (171). In addition, a chromatin immunoprecipitation study showed that ICAM1 expression by TNF-α is due to changes in the epigenome. In this study, TNF-α activated ICAM1 expression by initiating demethylation at lysine 9 and 27 of histone H3 (H3K9 and H3K27). Furthermore, the inhibition of enzymes G9a and EZH2, which are H3K9 and H3K27 methylators, showed the influence of G9a during ICAM1 expression. The observed action of KDM4B, a histone demethylase targeting H3K9me2, further highlights the importance of epigenetic regulation during TNF-α induced ICAM expression (172). These epigenetic factors similarly affected VCAM1 expression by TNF-α. In addition, VCAM1 expression in heart tissues was found to be dependent on STAT3 acetylation and GAT6 promoter methylation (173). MAdCAM1 is primarily released in response to gut inflammation, and the epigenetic regulation underlying its expression is still unclear. However, since MAdCAM1 expression is also triggered by TNF-α binding, there is a high potential for epigenetic regulation of its expression (174).

Leucocyte recruitment by adhesion molecules is critical during TLS formation. Hence, therapeutic options ensuring controlled, cell-specific expression of these molecules is essential. Specific modulation of the epigenetic changes involved in VCAM1, ICAM1, and MAdCAM1 release could be an effective strategy.

#### 3.4.6 High endothelial venules

HEVs are specialized blood vessels that aid lymphocyte migration to the lymphoid organs. They are in the lymph nodes, TLS (Extranodal HEV), and all SLOs except the spleen. The interaction between chemokines, adhesion molecules, and extranodal HEV facilitates the recruitment of blood-borne lymphocytes, which is vital in the development and immune function of the TLS (175). The presence of HEV at tumor sites has been linked to therapy success and reduced tumor growth. Other studies implicate HEV as an exit route for cancer progression and metastasis (176, 177). This is why many studies have tried to understand the mechanisms behind HEV neogenesis and function. However, the lack of markers to identify the developmental stages of these venules limits the knowledge of HEV formation (178, 179). Ligand binding to LT-BR was observed as a critical step towards HEV formation. Barring these limitations, the factors necessary for the lymphocyte trafficking function of HEVs have been well characterized. Expression of sulfated and glycosylated L-selectin ligand protein [called peripheral node addressins (PNAd)] by HEVs is an essential requirement for this function (180). PNAd proteins include endomucin, GlyCAM-1, CD34, and nepmucin (178). The interaction of PNAd with L-selectin on lymphocytes influences lymphocyte tethering to the HEV and their migration to the TLS or SLO. Therapeutic strategies focused on HEVs might involve stimulating the expression of PNAd in HEVs or L-selectin on lymphocytes. The expression of PNAd on the endothelial cells of HEVs was found to be regulated by the TF, DACH1 (181). DACH1 downregulation due to hypermethylation at its promoter region enhanced the growth and progression of gastric, colorectal, breast, and hepatocellular carcinoma (182–185). Specific demethylating agents at the DACH1 promoter region could stimulate its expression, activating PNAd expression and HEV formation.

These findings confirm that epigenetic reprogramming could be used to induce the expression of DACH1 and PNAd to cause an increase in HEV immune trafficking for TLS development.

## 4 Biomarkers of TLS

TLS comprises a spectrum of cell types, ranging from dispersed T-cell and B-cell aggregates to intricately organized formations of distinct T-cell zones and B-cell regions housing GCs. Consequently, the selection of potential markers is influenced by this variability. The development of TLS is considered a progressive phenomenon; however, conducting an in-depth analysis of TLS phenotypes and discerning the clinical significance associated with the maturation stages of TLS poses a significant challenge. Evidence suggests that analyzing the presence of immune cells, spatial distribution, and interplay within cancer sites could yield further valuable information. For example, patients suffering from HCC or CRC, whose TLS exhibited characteristics similar to primary or secondary follicles, demonstrated a decreased risk of recurrence in contrast to those with lymphoid clusters (186, 187).

Concerning TLS location, studies have not found differences in prognostic benefits between the peri- or intratumoral TLS. Both formations have been described in HCC (186), germ cell tumors (188), and lung metastases originating from renal carcinoma (189). However, in HCC, peritumoral presence compared to intratumoral has even been associated with an increased risk of recurrence and unfavorable prognosis (186). Numerous studies question the predictive role of TLS. These findings imply that the predictive influence of TLS might result from factors beyond their quantity and composition, encompassing the functionality and activity of the cells contained within them.

Concerning TLS composition, in broad terms, TLS exhibits an internal arrangement characterized by a central zone housing CD20+ B cells, encircled by a perimeter of CD3+ T cells, reminiscent of the lymphoid follicles observed in secondary lymphoid organs (SLOs) (10, 190, 191). As previously described, the trafficking of lymphocytes and dendritic cells (DC) to and within TLS is orchestrated by lymphoid chemokines, akin to the mechanisms observed in secondary lymphoid structures such as lymph nodes. Within the T-lymphocyte subset, CD4+ T follicular helper (T_FH_) cells frequently emerge as a predominant faction (192), purportedly capable of instigating TLS formation. However, a diverse repertoire is found within this compartment, including Th1 cells, CD8+ cytotoxic T cells, and regulatory T cells (15, 193).

T_FH_ cells represent a distinct subset facilitating T cell-mediated support for B cells. These cells predominantly reside within B cell follicles; specific GC is present in SLOs and TLS. Notably, B cells exhibit robust expression of C-X-C chemokine receptor 5 (CXCR5), facilitating their interaction with CXCL13, thereby guiding their migration towards follicular dendritic cells to establish germinal centers (194). Consequently, the surface expression of CXCR5 on T_FH_ cells acts as an attractant, directing their movement into B cell follicles and enabling intimate cell-cell contact with B cells. T_FH_ expresses a diverse repertoire of other surface molecules, such as programmed death-1 (PD-1) or B cell lymphoma-6 (BCL-6), which is the primary transcriptional factor of T_FH_ (195). TLS harbor diverse populations of DC, exemplified by the presence of CD21^+^ CD23^+^ follicular dendritic cells (FDC), as demonstrated by several authors (11, 15). These FDCs, arising from a mesenchymal origin, are pivotal in selecting memory B cells within GC reactions occurring in SLO.

When determining the appropriate markers to identify TLS, it is crucial to carefully consider the existence of three distinct classes of TLS that have been delineated. Some authors describe a primary class of *immature TLS (iTLS)*, characterized as accumulations of T and B cells with limited DC presence. Such structures lack definitive evidence of eliciting robust immune responses. They are notably associated with a context of T-cell depletion, inflammation, or an immunosuppressive TME, as observed in conditions such as HCC (196) and luminal BC (197). In luminal BC accompanied by *iTLS*, the T cell population exhibits features reminiscent of an exhausted phenotype, displaying modified cytotoxic attributes. Concurrently, the tumor cells had diminished expression of major histocompatibility complex class I (MHCI) molecules, implying a plausible deficiency in immune evasion mechanisms. The second and third categories incorporate *mature TLS (mTLS)*, which includes a B cell compartment forming either a primary follicle-like structure (PFL) or an SLO housing germinal centers (GCs). Within mTLS, mature dendritic cells establish contact with T cells, and a population of CD4^+^ PD-1^+^ CXCR5 ^+^ T_FH_ cells interact closely with B cells (198).

There is no consensus on when to consider TLS as mature. As mentioned above, most agree that the expression of CD20+ B cells should be present. The lack of agreement arises when selecting additional markers. Some authors suggest TLS reaches maturity when they present CD23+ or CD21+ FDC (11, 198, 199), resembling the description of a mature secondary lymphoid follicle. However, authors like (15) argue that an immature TLS should minimally exhibit CD23+ or CD21+ FDCs and propose that maturity is achieved upon the formation of GC, marked potentially by BCL-6+ in B cells (192, 198), DC-Lamp+ mature dendritic cells and CD23+ FDC (200). Others do not describe maturity without markers such as CXCR5+, CXCL13+, or even positive cellular proliferation markers like Ki67+ (10).

As previously mentioned, the CD20+ B cell zones harbor a network of CD21+ CD23+ FDC embedded within a structure resembling the PFL, considering that they are mature TLS in these cases. Of notable importance is the early presence of PNAd+ positive HEV, specialized blood vessels involved in lymphocyte trafficking, which may play a crucial role in initiating and maintaining TLS (198, 201). In addition, PNAd expression on the intratumoral vasculature was higher in tumors that expressed a well-presented CD8+ T cell antigen (138). These vessels develop in inflamed tissues and could potentially promote the formation of T cell aggregates, facilitating lymphocyte entry into tumor sites. On top of that, some authors, such as (31), have demonstrated that cancer-associated fibroblasts (CAF) can induce the formation of TLS by creating a reticular network that facilitates the influx of T and B cells through HEV. CD8+ T lymphocytes further mediate their function. The presence of CAF can be identified through the marker podoplanin (PDPN) (202), which, when highly expressed, is associated with CD140a and fibroblast activation protein (FAP). Therefore, the surface expression of these indicators may precede the development of tumor-associated lymphoid structures. A heightened expression of CXCL13 is a common occurrence in TLS (10, 203, 204), and it has been demonstrated that fibroblasts within inflamed tissues where TLS exhibit an extensive expression of this molecule (203, 205).

**Figure 3:**
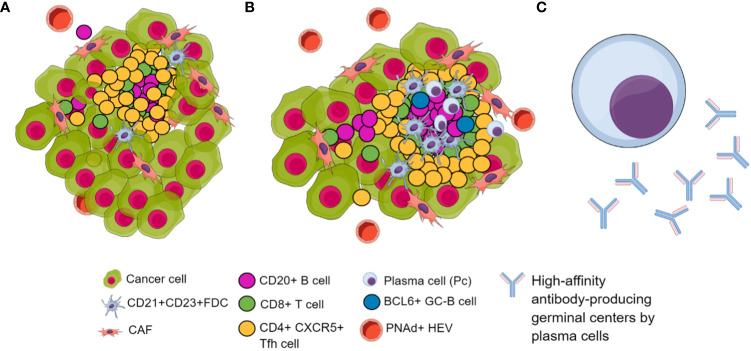
Biomarkers of TLS in their different maturation stages. **(A)** Immature TLS. **(B)** Mature TLS characterized by a higher infiltration of tumor-associated lymphocytes featuring follicular dendritic cells. Additionally, it features BCL6+ B cell attractors of T_FH,_ inducing lymphoid follicle formation with germinal centers. Additionally, there is an increase in PNAd+ HEVs, facilitating immune cell trafficking. **(C)** Long-lived, high-affinity antibody-producing plasma cells differentiated within GC of lymphoid follicles included in TLS.

In conclusion, despite the absence of a marker panel to determine TLS maturity, it is imperative to consider the distinct evolutionary stages of TLS and the specific tumor type under investigation. Consequently, the pan-B-cell marker CD20 must have been studied to perform a B-cell phenotyping that includes plasma cells using CD138 and CD19 (190). It is essential to utilize markers for FDC, such as CD21 or CD23, and assess the presence of PNAd+, indicating the establishment of HEVs. Likewise, known molecular markers of TLS formation include increased expression of CXCR5 and CXCL13, and their presence could be essential because their expression facilitates and guides migration toward TLS formation and the production of high-affinity antibody-producing germinal centers. Additionally, together with CXCR5, examining CD4, BCL6, and PD-1 could elucidate the presence of T_FH_ cells and a combination of FoxP3 and CD25 markers could be used to differentiate T_FH_ cells (CD25-FoxP3-) from regulatory T cells (CD25+, FoxP3+). Furthermore, CD8 markers have been associated with TLS in different tumor scenarios. Interestingly, marking other non-immune cells in the TLS, such as cancer-associated fibroblasts (CAF) networks using PDPN or FAP, may serve to detect TLS in earlier stages of formation as well as the stromal component of TLS (206) (**Figure 3**).

Although it has been suggested that the variation in the maturity level or positioning of TLS might impact their prognostic significance, further studies are needed to define precise markers regarding the stages and location of TLS. Accordingly, the primary challenge in comprehending the impact and importance of TLS in the antitumoral response of patients lies in the lack of consensus regarding the definition of their composition and how the quantification is performed in the laboratory, notably, the methodology to be employed for their characterization (207). This complexity significantly impedes the use of the presence, composition, and density of TLS as a standard clinical marker. For all these reasons, it is necessary to develop a robust, reproducible, and reliable diagnostic tool that allows pathology laboratories to study TLS presence in diagnostic biopsies directly across all tumor types

## 5 Discussion

In clinical practice, TLS have been associated with improved response to immunotherapy and favorable prognosis. This correlation has resulted in considering TLS as a putative biomarker for immunotherapy response and prognosis (208). Throughout this review, the critical features for both TLS induction and detection have been highlighted, and the general conclusion is that specific cytokines like CXCL13, follicular markers such as CXCR5, and epigenetic regulatory mechanisms affecting cornerstone TF that stimulate the different pathways of TLS formation constitute promising biomarkers and therapeutic targets.

Lentivirally transduced CAR-T cells or DC stand at the forefront of the *in vivo* manipulation of the TME. IL-7 and CCL21 are examples of relevant transgenes used to induce TLS in these systems. In the context of the recruitment and binding of LTi cells expressing lymphotoxin- α1β2 (LT- α1β2) to stromal cells, the co-expression of IL-7 and CXCL13 would be a further relevant approach, especially considering that CXCL13 is involved in various processes necessary for TLS establishment and an immunoreactive TME (B cells chemoattraction and GC formation, HEV development). Moreover, since LVs are amenable to CRISPR-mediated genome editing, specific genetic and epigenetic variations could be tested for efficacy of TLS formation. In this regard, we have identified epigenetic reprogramming as a strategic approach. On the one hand, there is an exciting transdifferentiation phenomenon related to TLS. Several cell types, such as ILC3, Th17, and even NK, CD8, B cells, and macrophages, are classified as putative surrogates of the TLS-initiating cells LTis. Manipulation of the TF involved in their fate determination is a plausible strategy where several epigenetic modifications take part. Moreover, NF-Kβ regulates TLS formation at multiple levels, from ligand binding to LT-βR to the expression of integrin ligands important for leucocyte recruitment. Although the double-edge functions of this TF can limit its applicability, several activators and inhibitors are available and could be tested. Another important TF in this process is TNF-α, which has been extensively reported to be regulated by DNA methylation and histone acetylation. Finally, PNAd, a major inducer of the formation of HEV, has already been overexpressed using a demethylating agent. Of note, PNAd methylation correlates with tumor growth and progression.

The biomarker perspective in the TLS field is also controversial since there are multiple markers of mature TLS, but none have been clinically validated. In this regard, there are several aspects worth consolidating. One refers to the absence of segment correlations per stage of maturation. Establishing a consensus signature of a TLS phenotype that is of predictive and prognosis value is an essential but complex task. The second aspect that relates to this is the need to implement high-resolution techniques to characterize the cellular composition, interactions, and spatial distribution of the TLS quantitatively and qualitatively. Single-cell sequencing, spatial transcriptomics, and multispectral immunofluorescence can serve this cause and promote the identification of feasible immunohistochemistry markers for clinical use.

## Author contributions

QAO: Conceptualization, Data curation, Investigation, Methodology, Writing – original draft, Writing – review & editing, Visualization. AE: Data curation, Investigation, Methodology, Visualization, Writing – original draft. CF: Conceptualization, Investigation, Methodology, Writing – original draft. EP-R: Project administration, Resources, Supervision, Validation, Writing – review & editing. AR-D: Project administration, Resources, Supervision, Validation, Writing – review & editing, Conceptualization, Funding acquisition, Investigation, Methodology. IB: Conceptualization, Funding acquisition, Investigation, Methodology, Project administration, Resources, Supervision, Validation, Writing – review & editing, Data curation, Writing – original draft.

## Glossary

**Table:**

**Abbreviations**	**Meaning**
**TME**	Tumor Microenvironment
**TLS**	Tertiary Lymphoid Structures
**TILs**	Tumor-infiltrating lymphocytes
**CAR**	Chimeric Antigen Receptors
**ICI**	Immune checkpoint inhibitors
**SLOs**	Secondary Lymphoid Organs
**HCC**	Hepatocellular carcinoma
**LTi**	Lymphoid tissue-inducer cells
**APC**	Antigen-presenting cells
**IL-7**	Interleukin-7
**LT-α**	Lymphotoxin-α
**LT-βR**	Lymphotoxin-β receptor
**CXCL13**	CXC-chemokine ligand 13
**CXCL12**	CXC-chemokine ligand 12
**CCL19**	CC-chemokine ligand 19
**CCL21**	CC-chemokine ligand 21
**LT- α1β2**	Lymphotoxin- α1β2
**VEGFC**	Vascular endothelial growth factor C
**VCAM1**	Vascular cell adhesion molecule 1
**MAdCAM1-**	Mucosal addressin cell-adhesion molecule 1
**ICAM1**	Intercellular adhesion molecule 1
**HEV**	High endothelial venules
**GC**	Germinal centers
**VCAM1**	vascular cell adhesion molecule 1
**NK cells**	Natural Killer Cells
**ILC3**	Innate Lymphoid Cell 3
**CAF**	Cancer-Associated Fibroblast
**TNF- α**	Tumor Necrosis Factor- α
**PDAC**	Pancreatic ductal adenocarcinoma
**T-bet**	T-box transcription factor TBX21
**HAT**	Histone acetyltransferases
**TET**	Ten-eleven translocation
**HDM**	Histone demethylases
**HA**	Histone acetylation
**CTCL**	cutaneous T-cell lymphoma
**EZH2**	Enhancer of zeste homolog 2
**LSD**	lysine demethylase
**ncRNA**	Non-coding RNA
**lncRNA**	long non-coding RNA
**RNAi**	RNA interference
**miRNA**	microRNA
**T-UCR**	transcribed ultraconserved regions
**HOTAIR**	HOX antisense intergenic RNA
**EMT**	epithelial-to-mesenchymal transition
**ILC1, ILC2, ILC3**	Innate lymphoid Cells 1,2,3
**ftILCP**	fetal transitional Innate Lymphoid Cell Precursor
**CHILP**	common helper innate lymphoid progenitor
**NFκB**	nuclear factor κB
**JNK**	c-Jun N-terminal kinase
**IκB**	Inhibitor of κB
**NIK**	NF-Kappa-B-inducing kinase
**MAP3K**	Mitogen-activated protein-3-kinase
**ECs**	Endothelial Cells
**BC**	breast cancer
**CRC**	Colorectal Cancer
**PNAd**	peripheral node addressins
**NSCLC**	non-small cell lung cancer
**T_FH_ Cells**	T follicular helper cells
**PD-1**	programmed death-1
**BCL-6**	B cell lymphoma-6
***iTLS***	Immature *TLS*
***mTLS***	Mature *TLS*
**PDPN**	Podoplanin
**FAP**	Fibroblast activation protein
**BCL-6**	B cell lymphoma-6
**TA-TLS**	Tumor Associated TLS
**FDC**	Follicular Dendritic Cells
**FRC**	Follicular Reticular cells
**DC**	Dendritic cells
**MSC**	Mesenchymal Stem Cells
**AAV**	Adenoviral vector
**LV**	Lentiviral vectors
**TF**	Transcription Factors
**DNMT**	DNA methyltransferases
**HMT**	Histone methyl transferases
**HDAC**	Histone deacetylases
**TSG**	Tumor suppressor gene
**SAHA**	suberoylanilide hydroxamic acid
**PTCL**	peripheral T cell lymphoma
**TCP**	tranylcypromine
**ATRA**	all-trans-retinoic acid
**sncRNA**	Small non-coding RNA
**piRNA**	PIWI-interacting RNA
**snoRNA**	small nucleolar RNAs
**endo-siRNA**	endogenous small-interfering RNA
**lincRNA**	long intergenic non-coding RNA
**MEG 3**	maternally expressed 3
**ASO**	antisense anti-oligonucleotides
